# Accuracy of a helium‐beam radiography system based on thin pixel detectors for an anthropomorphic head phantom

**DOI:** 10.1002/mp.17786

**Published:** 2025-03-26

**Authors:** Margareta Metzner, Friderike K. Longarino, Benjamin Ackermann, Annika Schlechter, Maike Saphörster, Yanting Xu, Julian Schlecker, Patrick Wohlfahrt, Christian Richter, Stephan Brons, Jürgen Debus, Oliver Jäkel, Mária Martišíková, Tim Gehrke

**Affiliations:** ^1^ Heidelberg Institute for Radiation Oncology (HIRO) and National Center for Research in Radiation Oncology (NCRO) Heidelberg Germany; ^2^ German Cancer Research Center (DKFZ) Heidelberg Division of Medical Physics in Radiation Oncology Heidelberg Germany; ^3^ Department of Physics and Astronomy Heidelberg University Heidelberg Germany; ^4^ German Cancer Research Center (DKFZ) Heidelberg Clinical Cooperation Unit Translational Radiation Oncology Heidelberg Germany; ^5^ Department of Radiation Oncology Heidelberg University Hospital Heidelberg Germany; ^6^ Heidelberg Ion Beam Therapy Center (HIT) Department of Radiation Oncology Heidelberg University Hospital Heidelberg Germany; ^7^ EP Department CERN Geneva Switzerland; ^8^ German Cancer Research Center (DKFZ) Heidelberg Division of Radiooncology/Radiobiology Heidelberg Germany; ^9^ OncoRay ‐ National Center for Radiation Research in Oncology Faculty of Medicine and University Hospital Carl Gustav Carus Technische Universitat Dresden, Helmholtz‐Zentrum Dresden‐Rossendorf Dresden Germany; ^10^ Helmholtz‐Zentrum Dresden‐Rossendorf Institute of Radiooncology ‐ OncoRay Dresden Germany; ^11^ Department of Radiotherapy and Radiation Oncology Faculty of Medicine and University Hospital Carl Gustav Carus Technische Universität Dresden Dresden Germany; ^12^ German Cancer Consortium (DKTK) Partner Site Dresden Dresden Germany; ^13^ National Center for Tumor Diseases (NCT) NCT Heidelberg, a partnership between DKFZ and Heidelberg University Hospital Heidelberg Germany; ^14^ German Cancer Research Center (DKFZ) Heidelberg Clinical Cooperation Unit Radiation Oncology Heidelberg Germany

**Keywords:** ion‐beam imaging, proton therapy, timepix detectors

## Abstract

**Background:**

Ion‐beam radiography is a promising technique to verify the range of ion‐beam radiotherapy treatments regularly. To detect and quantify the water‐equivalent thickness (WET) of potential anatomical changes, ion‐beam radiographs must provide a sufficient WET accuracy on the level of 1%.

**Purpose:**

In this work, we show an energy‐painted helium‐beam radiograph of an anthropomorphic head phantom acquired with thin silicon pixel detectors for the first time. Furthermore, we determine the WET accuracy of our helium‐beam radiography system for the especially heterogeneous skull base region, which is highly relevant for the treatment of head and neck and skull base tumors.

**Methods:**

With a detection system based on pixelated semiconducting Timepix detectors, we track single ions upstream and downstream of the head phantom. Furthermore, we measure their energy deposition in a thin Timepix detector behind the anthropomorphic phantom. To ensure a high precision of the image, we acquired a radiograph by using helium beams with five initial energies between 146.84 and 188.07 MeV/u following the energy painting algorithm. With a Siemens SOMATOM Confidence CT scanner, a single‐ and dual‐energy CT were acquired with clinical protocols and translated to relative stopping power (RSP) values. After projecting these scans, the resulting WET maps were compared to the helium‐beam radiograph. To evaluate the accuracy of all three modalities, a reference data set based on range‐pullback measurements and a segmentation of a high‐resolution CT scan was taken into account.

**Results:**

The mean absolute percentage error (MAPE) of all modalities was determined to be between 0.95% and 1.16%. Also, the root‐mean‐square percentage error (RMSPE) was similar for all modalities ranging from 1.19% to 1.46%. These deviations from the reference scan were found to mainly stem from an overestimation of air and sinus tissue and underestimation of cortical bone.

**Conclusions:**

The helium‐beam radiograph was shown to achieve a WET accuracy competitive with that of clinically used imaging methods. If certain technical aspects are addressed, helium‐beam radiography may emerge as an auspicious imaging modality for on‐couch range verification of ion‐beam radiotherapy treatments allowing for regular detection and quantification of anatomical changes.

## 1 INTRODUCTION

Radiation therapy is an important modality for cancer treatment. Conventionally, photons are used to irradiate cancer cells. Ion‐beam radiotherapy is an extremely promising alternative due to the fundamentally different interactions of ions with matter. The steep increase of dose at the end of the range of ions, the so‐called Bragg peak,^1^ can be utilized to create more focused dose distributions compared to conventional treatments with photons.^2^, ^3^, ^4^, ^5^, ^6^ Lower dose deposited in healthy tissue is expected to lead to fewer side effects and occurrences of secondary tumors.^7^

However, the steep gradients in the focused dose distribution lead to an increased sensitivity of the treatment to uncertainties. Small changes can have a major impact on the tumor control or normal tissue complications. Therefore, the mitigation of uncertainties is of major importance for ion‐beam radiotherapy.^8^, ^9^

To calculate the dose distribution precisely, the interactions of the ions with the tissue need to be known for each patient. The important physical quantity for this, the relative stopping power (RSP)^a^ of the tissue, is usually obtained by means of x‐ray computed tomography (CT) images. The quantitative x‐ray CT numbers (CTN), which are closely related to photon attenuation coefficients, are translated to RSP values via a CTN‐to‐RSP lookup table.^10^ However, these translations can lead to uncertainties in the calculation of the ions' range in patient tissue of about 2.3% – 2.6% (1.5 σ).^11^, ^12^ This is due to the fact that the two quantities, which reflect two fundamentally different physical processes, are not connected via a unique relation.

Another effect contributing to the range uncertainties is the planning CT images only being able to capture the anatomy of the patient before the treatment starts. If the patient anatomy changes, for example, due to the filling of a cavity, the delivered dose will look different from the planned one. Depending on the size of the anatomical change, the range uncertainties originating from this can even be more severe than the ones based on the CT uncertainties.

To validate ion‐ treatment plans regularly, potentially on a daily basis, low‐dose ion‐beam radiography is a very promising technique. If these ion‐beam radiographs are quantitative in terms of water‐equivalent thickness (WET), which equals integrated RSP along beam direction, they may be compared to projections of the planning x‐ray CT. Consequently, uncertainties due to inter‐fractional changes of the patient's anatomy relevant for the treatment could be identified as well as errors stemming from the CTN to RSP translation.^9^

Several studies have investigated ion CT performance for treatment planning purposes and compared their RSP or WET uncertainties in geometric and anthropomorphic phantoms with conventional x‐ray CT imaging modalities experimentally.^13^, ^14^, ^15^, ^16^, ^17^

In this work, we investigate the WET accuracy of a helium‐beam radiograph (αRad) since it has been previously shown to be superior to proton radiography in terms of spatial resolution.^18^ Similar to refs. [19, 20, 21, 22], we use multiple initial beam energies for the acquisition of a radiograph. The energy painting approach together with a small and light imager based on thin silicon pixel detectors was proven to yield very accurate and precise WET values in a geometric phantom.^23^ In the work at hand, the energy painting is applied to acquire a helium‐beam radiograph of an anthropomorphic phantom with Timepix detectors for the first time. To assess the WET accuracy for the anthropomorphic head phantom,^24^ a reference WET‐map based on the segmentation of a high‐resolution CT scan and range‐pullback measurements of all materials contained in the phantom is used.

Since such a reference WET‐map is only available for phantoms, we also compare our WET accuracy to clinically established methods available for imaging of patient tissue. For daily WET verification, cone‐beam CT has been proposed and numerous different methodologies have been investigated in studies.^25^ However, no method is widely spread and established as a clinical standard yet,^26^ which makes a comparison difficult. Instead, we compare our WET accuracy with CT modalities, which are used for treatment (re‐)planning, and therefore, also deposit a higher dose than daily imaging modalities. However, their use in clinics is well established and therefore a comparison with these modalities is more informative.

## 2 MATERIAL AND METHODS

### 2.1 Anthropomorphic phantom and its reference data set

To make the assessment of WET accuracy in an anthropomorphic phantom feasible, a ground truth WET‐map is required. For this purpose, the reference RSP map previously published by Wohlfahrt et al.^27^ was used, which will be referred to as the “reference” in this work.

This map was established for the 731‐HN phantom (Proton Therapy Dosimetry Head, Model 731‐HN; CIRS, Norfolk, VA), a head phantom made from epoxy resin, which is tissue equivalent for both photons and protons.^24^ Wohlfahrt et al.^27^ determined the RSP values of all nine tissue surrogate materials occurring in the phantom by range‐pullback measurements of homogeneous slabs provided by the manufacturer. Furthermore, they acquired a high‐resolution CT scan of the phantom (voxel size (0.5 mm)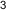), performed segmentation and assigned range‐pullback RSP values. They also validated the RSP map for verification of RSP and range “with a conservative upper limit of 0.3% and 1 mm uncertainty, respectively, even in the most complex inhomogeneous case.”^27^

For all experiments, the head phantom was positioned on a clinical treatment pillow and a head step.

### 2.2 X‐ray CT image acquisitions

The x‐ray‐based single‐energy CT (SECT) and dual‐energy CT (DECT) images of the anthropomorphic head phantom were acquired using a SOMATOM Confidence CT scanner (Siemens Healthineers, Forchheim, Germany). The scanner uses a sequential DECT acquisition technique (dual spiral), scanning the entire volume sequentially at two different tube potentials. Both the SECT and DECT scans were performed with state‐of‐the‐art clinical head protocols.^28^ The following SECT (DECT) image acquisition settings and reconstruction parameters were used: tube voltage of 120 kV_p_ (80/140 kV_p_), tube current‐time product of 165 mAs (248/58 mAs), collimation of 2 × 32 × 0.6 mm, rotation time of 1 s (0.5 s), pitch of 0.55, CTDI_vol_ of 24.9 mGy (11.1/12.7 mGy) using 32 cm CTDI_vol_ diameter, voxel size of $0.9766\times 0.9766\times 1.5m^{3}$, and Qr40 reconstruction filter with bone beam hardening correction. The RSP prediction for the SECT image data was based on a clinically employed Hounsfield lookup table (HLUT), a calibration procedure used to obtain RSP from CT numbers.^10^, ^29^, ^30^ For DECT imaging, a DirectSPR implementation^31^ was used in the syngo.via image reconstruction software (Siemens Healthineers, Forchheim, Germany).

### 2.3 Ion‐beam radiography experiments

The reported ion‐beam radiography experiments were carried out at the Heidelberg ion‐beam therapy center (HIT).^32^, ^33^ Because helium ions (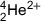) were shown to provide a better spatial resolution compared to protons and better statistics in each pixel at the same dose compared to carbon ions,^18^ they were used for all ion imaging experiments. In order to be able to use the energy painting approach,^23^ five initial beam energies were chosen between 146.84 and 188.07 MeV/u with a focus size of 10.3 – 11.1 mm (FWHM) and intensities of around $10^{4}$–$10^{5}$ particles/s.

The ion‐beam radiography imager used in this work is based on six first generation Timepix detectors.^34^ Our hybrid detectors^b^ consist of a Timepix chip and a 300 μm‐thin sensitive layer made from silicon. The Timepix detectors have a sensitive area of 14 mm × 14 mm and a pixel pitch of 55 μm in both dimensions.^34^

**FIGURE 1:**
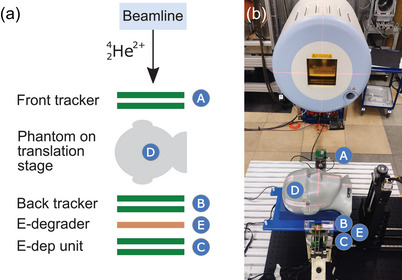
Helium‐beam radiography system^35^ based on Timepix detectors.^34^ The detection system, which is shown in (a) schematically and (b) as a photograph, was aligned with the 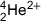
‐beam in the experimental room. Upstream of the imaged object (D), the front tracker (A) is positioned. The back tracker (B), the energy degrader (E) and the energy deposition unit (C) are placed downstream of the phantom.

Six of these detectors are arranged along the beam axis,^35^ as depicted in Figure 1, and synchronized with a dedicated hardware unit. Two detectors each are operated by one readout unit called fitPix^36^ and form one detection unit. One of these units is positioned upstream of the imaged object (A) and two downstream (B,C). Unit (A) and (B) are the so‐called tracking units and all detectors within these units are configured to measure in time of arrival (ToA) mode. In unit (C), the first detector measures the time over threshold (ToT), which is closely connected to the energy deposition and used to produce the image contrast.

The first generation Timepix detectors operate in so‐called “frame mode,” which means that each measurement time, called frame, is followed by a dead time during which all pixels are read out. The frame duration was set to 1 ms, which is followed by around 30 ms dead time. Because a synchronization of the active time with the beam is conceivable and the next detector generation, Timepix3, can be operated dead‐time free, we only report the dose on our detector during the active time, which actually contributed to the image.

The energy degrader (E)^35^ was introduced to improve the spatial resolution of the system and to also ensure that there is some material between the phantom—or later patient—and the high energy deposition on the last detection unit for future clinical applications.

The phantom (D) was aligned between the two tracking units by means of markings and metal stickers on the phantom together with the laser system of the experimental room to position the phantom in an orientation as similar as possible to the one in the x‐ray CT scans described in Section 2.2.

**FIGURE 2:**
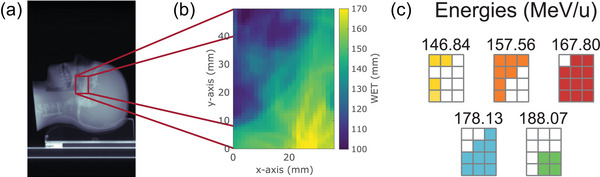
Region and energy selection based on single‐energy CT (SECT). (a) Projection of a SECT scan and imaged region marked in red. The CT numbers were simply summed up to obtain the usual x‐ray CT contrast. (b) In the zoomed‐in region, the SECT was converted to RSP values first (see Section 2.2) and was consecutively projected to 2D. In this way, a quantitative WET map was obtained. (c) The initial helium‐ion energies, which were used to image the different ROIs, were selected based on the WET interval that we expected for each ROI in the x‐ray CT. CT, computed tomography; ROIs, region of interest; RSP, relative stopping power; SECT, single‐energy computed tomography; WET, water‐equivalent thickness.

The imaging region of the anthropomorphic phantom was chosen to contain heterogeneous tissue materials in a clinically relevant treatment area. The selection was based on the SECT scan. Since the envisaged clinical scenario would be a daily verification of the patient anatomy with one or several radiographs, the planning SECT would be available for each patient and could serve for that purpose. The chosen region, which is shown in Figure 2a and has dimensions of 48 mm × 36 mm, was divided into 12 regions of interest (ROIs), all with the size of 12 mm × 12 mm to make imaging with the given field of view (FOV) of the detector feasible. The images were acquired subsequently, after a lateral translation of the phantom between the fixed tracking units. The data of all ROIs was later combined into the final image by a modification of the tracks using the known conducted shifts of the translation stage.^23^

Because the imaged region of the phantom was expected to have WET values between approximately 90 and 180 mm, a high‐precision image cannot be acquired with only one initial beam energy using this experimental detection system. Our helium‐ion imager is based on the measurement of energy deposition in thin detectors instead of the conventionally measured residual energy or range.^8^, ^37^, ^38^ Therefore, we applied the previously developed energy painting algorithm^23^ to always keep the steep rising edge of the Bragg peak in the volume of the relevant detector. Following this algorithm, five initial beam energies were used to image different ROIs as depicted in Figure 2c to image the WET range of 90 to 180 mm with high precision.

In order to reconstruct helium‐beam radiographs from the measured data, the post‐processing routines described in Metzner et al.^23^ and dE‐WET‐calibrations established in Knobloch et al.^39^ were applied. For the post‐processing, only a subset of the measured data was used in order to reconstruct a radiograph with a clinically relevant dose.

### 2.4 Image registration and projection of 3D data sets

As in clinical routine, markings and metal stickers were attached to the phantom to identify its position in the planning SECT. In this way, a rough hardware registration to the planning SECT is possible for each irradiation session in clinics. In our case, using a rigid object, a reproducible positioning of the phantom for the imaging process can be achieved and the planned region and projection (see Section 2.3) can be imaged with small positioning uncertainties.

Because no markings of the scan for the reference RSP map of Wohlfahrt et al.^27^ were left on the phantom, their exact orientation of the phantom on the pillow could not be reproduced in our experiments. To obtain a connection to the reference, it was rigidly registered in 3D using MITK^c^ software to both the SECT and DECT image individually.^d^ As a similarity metric, Mattes mutual information^40^ was employed, and a regular step gradient descent was set as optimizer. The 3D data set of the reference was interpolated linearly to the grid of the SECT or DECT scan, respectively to obtain images with clinically used pixel sizes.

The registrations to the SECT and DECT image should theoretically yield the exact same result since the phantom was not moved between the scans. The two registrations were compared and a maximum displacement between both transformations of 0.1 mm was found, which is at least 10 times smaller than the pixel size of the CTs. Therefore, in very good approximation, the differences of the registrations can be assumed to be negligible. For further analyses only the reference, which was registered to the DECT, was used.

Air voxels outside of the phantom were removed in all 3D data sets to make a WET comparison of only the phantom feasible. Reference, SECT and DECT were then separately summed up along the sagittal direction and multiplied by the voxel size along that direction to obtain the discrete integral over the RSP values, which corresponds to the WET. For this discrete integration, the lateral dimensions were set to one pixel.

The measured helium‐beam radiograph was registered to the 2D WET map originating from the reference data set with MatchPoint software^41^ using Mattes mutual information as similarity metric and a linear interpolation to the pixel grid of the 2D reference image. Due to the interpolation during the image registration process, eventually all images have a pixel size of 0.98 × 1.5 ${\mathrm{mm}}^{2}$.

### 2.5 Metrics for quantitative comparison

To compare all imaging modalities to the reference, different metrics were used.

First, the relative WET difference ΔWET in each pixel p was calculated to obtain a 2D image showing the deviations from the reference image (R)

(1) $$\Delta \;\text{WET}\left(p\right)=\frac{\text{WET}\left(p\right)-{\text{WET}}_{R}\left(p\right)}{{\text{WET}}_{R}\left(p\right)}\;.$$

To compare the whole images consisting of in total P pixels (1≤p≤P) by using only one number, the mean absolute percentage error (MAPE)

(2) $$\text{MAPE}=\frac{1}{P}\sum_{p=1}^{P}\left|\Delta \;\text{WET}\left(p\right)\right|=\bar{\left|\Delta \;\text{WET}(p)\right|}$$

and the root‐mean‐square percentage error (RMSPE)

(3) $$\text{RMSPE}=\sqrt{\frac{1}{P}\sum_{p=1}^{P}{\left(\Delta \;\text{WET}(p)\right)}^{2}}$$

of the relative WET differences were determined. To be able to provide an estimate for the uncertainty of both MAPE and RMSPE, we divided the image into 42 sub‐regions and calculated both quantities for each of them. The standard error of the mean with respect to the MAPE or RMSPE of the whole image serves as our estimate for their uncertainty.

Furthermore, a local gamma analysis^42^ with 0.7% and 1 mm was conducted. The parameters reflect the uncertainties Wohlfahrt et al.^27^ stated for the reference scan: 1 mm delineation uncertainty and 1 mm in range, which corresponds to 0.7% for our mean WET of 135 mm.

## 3 RESULTS

**FIGURE 3:**
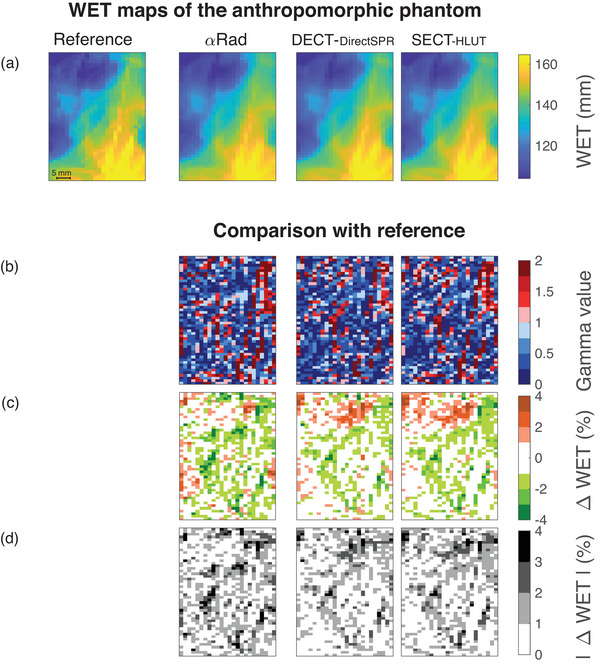
Quantitative comparison of the water‐equivalent thickness (WET) values determined by means of different imaging modalities. (a) The sagittal projection of a region at the skull base of the anthropomorphic phantom is shown quantitatively in terms of WET for the reference, the helium‐beam radiograph (αRad) and the radiographs based on dual‐energy CT (DECT‐DirectSPR) and single‐energy CT (SECT‐HLUT). (b) The gamma analysis^42^, ^43^ for 1 mm, 0.7% is depicted in comparison to the reference. The relative differences to the reference are displayed (c) with both negative and positive contributions and (d) in absolute terms such that deviations can be compared regardless of their sign. αRad, helium‐beam radiography; DECT, dual‐energy computed tomography; HLUT, Hounsfield look up table; SECT, single‐energy computed tomography; WET, water‐equivalent thickness.

The WET maps of all three imaging modalities are shown in Figure 3a together with a comparison to the reference. They are quantitative in terms of WET, which equals the integrated RSP and therefore is an important quantity for clinical treatment verification measurements. The images show the sagittal projection of a region at the base of the skull of the anthropomorphic head phantom (Section 2.1).

**FIGURE 4:**
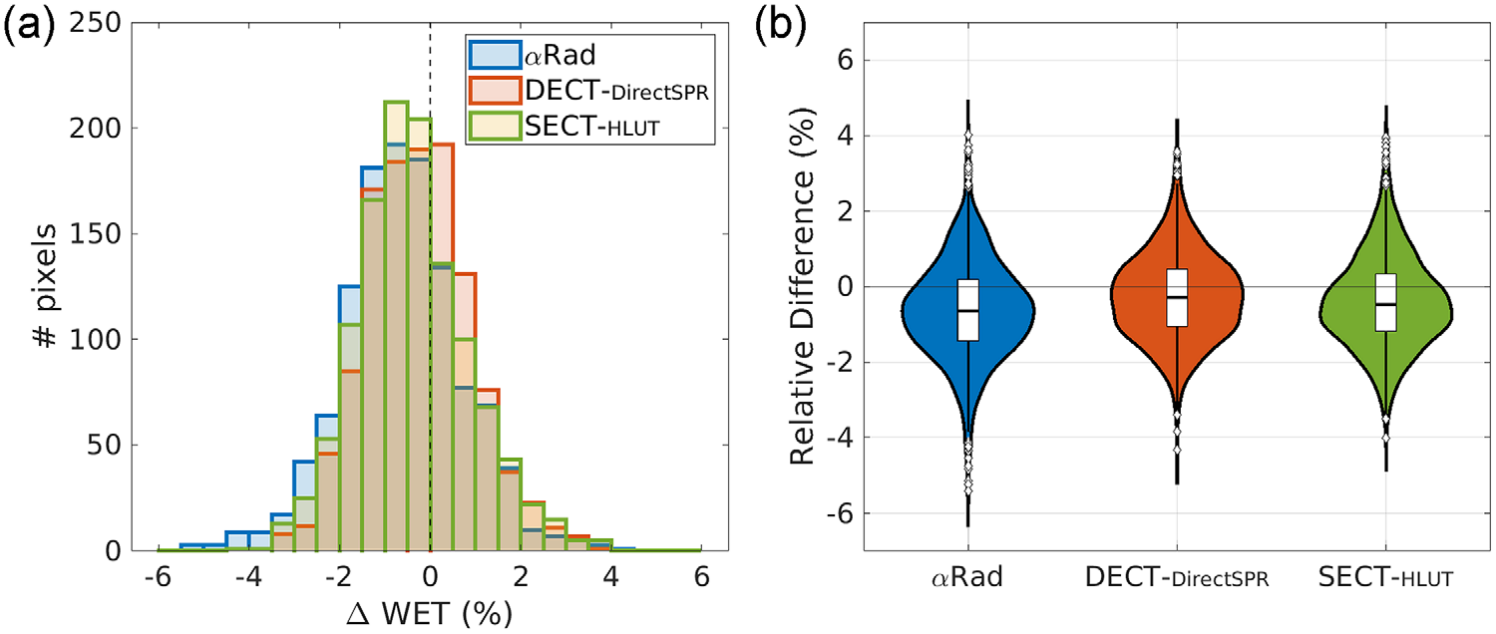
Analysis of relative differences in Figure 3c. (a) Histogram of WET differences relative to the reference for the αRad and the radiographs based on dual‐energy CT (DECT‐DirectSPR) and single‐energy CT (SECT‐HLUT). (b) Violin plot^44^ showing the relative WET differences with respect to the reference scan. The median value and the first and third quartile are additionally marked with the box plot. αRad, helium‐beam radiography; DECT, dual‐energy computed tomography; HLUT, Hounsfield look up table; SECT, single‐energy computed tomography; WET, water‐equivalent thickness.

In Figure 3b, the gamma analysis^42^, ^43^ with the parameters 1 mm and 0.7% is shown for the helium‐beam radiograph (αRad), the dual‐energy CT (DECT‐DirectSPR) and single‐energy CT (SECT‐HLUT) scan compared to the reference. Even under these strict conditions, passing rates of $\gamma_{\alpha \mathrm{Rad}}=\left(73.9\;\pm \;0.9\right)\;\%$, $\gamma_{\mathrm{DECT}}=\left(78.7\;\pm \;0.7\right)\;\%$, and $\gamma_{\mathrm{SECT}}=\left(74.1\;\pm \;0.8\right)\;\%$ were achieved. Figure 3c,d, respectively, show the relative and absolute percentage difference of the three modalities compared to the reference image. In Figure 4a, these relative differences are shown in a histogram and a violin plot.^44^ The standard deviations of the distributions are 1.33% (αRad), 1.16% (DECT‐DirectSPR), and 1.24% (SECT‐HLUT).

To determine a metric independent of the sign and to summarize the distributions by a single number, the MAPE and RMSPE were also calculated and are summarized in Table 1. The MAPE amounts to (1.16 ± 0.05)% for αRad, (0.95 ± 0.05)% for DECT‐DirectSPR, and (1.05 ± 0.06)% for SECT‐HLUT. The RMSPE is (1.46 ± 0.06)%, (1.19 ± 0.05)%, and (1.30 ± 0.06)%, respectively.

**TABLE 1:** Metrics for quantitative comparison of WET accuracy.

Metric	αRad	DECT‐DirectSPR	SECT‐HLUT
MAPE (%)	1.16 ± 0.05	0.95 ± 0.05	1.05 ± 0.06
RMSPE (%)	1.46 ± 0.06	1.19 ± 0.05	1.30 ± 0.06

*Note*: To summarize the distributions of relative WET differences by one number, the MAPE and RMSPE were calculated for the helium‐beam radiograph (αRad) and the radiographs based on dual‐energy CT (DECT‐DirectSPR) and single‐energy CT (SECT‐HLUT).

Abbreviations: αRad, helium‐beam radiography; DECT, dual‐energy computed tomography; MAPE, mean absolute percentage error; RMSPE, root‐mean‐square percentage error; SECT, single‐energy computed tomography; WET, water‐equivalent thickness.

**FIGURE 5:**
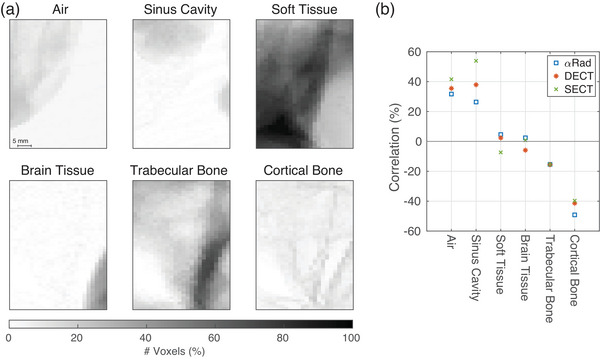
Correlation of WET differences with material abundance for the helium‐beam radiograph (αRad) and the radiographs based on dual‐energy CT (DECT‐DirectSPR) and single‐energy CT (SECT‐HLUT). (a) Shows the percentage of voxels along each pixel channel, which is made from a certain material. A pixel channel here is defined as the series of voxels in the reference data set over which the RSP was integrated to obtain the WET. (b) Shows the correlation of the three water‐equivalent thickness difference images in Figure 3c with the six images of Figure 5a. High (anti‐)correlations indicate that the deviations in Figure 3c are likely to be caused by the corresponding material. αRad, helium‐beam radiography; DECT, dual‐energy computed tomography; HLUT, Hounsfield look up table; RSP, relative stopping power; SECT, single‐energy computed tomography; WET, water‐equivalent thickness.

In Figure 3, we see that the relative difference with a high magnitude clusters in particular regions of the WET map. Since the WET in an anthropomorphic phantom always contains the RSP of several materials along the ions' paths, also the deviation of WET from the reference is caused by a combination of several materials. In Figure 5, we correlate the abundance of materials in each pixel channel with the deviation in WET from the reference. A pixel channel here is defined as the series of voxels in the reference data set over which the RSP was integrated to obtain the WET, so all voxels in the channel orthogonal to the image plane.

In Figure 5a, the same imaged region as in Figure 3 is shown. Instead of the WET, the color bar shows the percentage of voxels contributing with a certain material's RSP to each pixel's WET. Note that only voxels inside of the phantom were taken into account, which is why the percentage of voxels filled with air is low. In Figure 5b, the correlation between the relative difference maps in Figure 3c and the percentage maps in Figure 5a is depicted for αRad, DECT‐DirectSPR, and SECT‐HLUT. The correlation plot shows to which extent deviations in WET correlate with the abundance of a material in a channel. It can therefore be seen as a measure to determine by which material the deviations are caused. The highest correlation of the deviations was found for sinus cavity in SECT, which amounts to 54%. The deviations of all three modalities are strongly correlated with the abundance of sinus cavity as well as air and highly anti‐correlated to the one of bone, in particular cortical bone, αRad yielding the value with the highest magnitude of 49%.

## 4 DISCUSSION

In this work, we presented an energy‐painted helium‐beam radiograph of an anthropomorphic phantom acquired with thin pixel detectors. We investigated the WET accuracy of the radiograph and compared it to the one of clinically used x‐ray CT modalities. In this way, we demonstrated our system's capability to achieve comparable WET accuracy, while depositing two orders of magnitude less dose than the x‐ray CT scans in the patient revealing its potential as a powerful modality for daily WET verification.

We deliberately chose an imaging region at the base of skull, where many structures with high density changes overlap. The chosen region is clinically highly relevant for base of skull and head and neck tumors. Major anatomical changes can occur in the cavities there, for example, due to a cold, which makes it especially interesting for regular range verification. Additionally, this region is one of the most heterogeneous ones in a head phantom, which is why we expect this case to yield worse WET accuracy than more homogeneous regions, for example, within the brain. Therefore, we presented a conservative estimate of the capabilities of our imaging system in this work.

The mean dose of the αRad presented in this work was approximately (290 ± 190) μGy e. If the image is reconstructed with around 100 μGy, which corresponds to the same dose to the patient as an x‐ray positioning projection,^45^ the RMSPE will slightly increase to (1.52 ± 0.07)%. The authors consider this dose to be realistic for daily imaging. The CTDI_vol_ of the x‐ray CT scan was 24.9 mGy for SECT and 11.1/12.7 mGy for DECT. These two imaging modalities of course offer a way wider variety of applications, most importantly 3D information. However, for the very specific purpose of WET verification, which was investigated in this work, the helium‐beam radiograph performed similarly well with two orders of magnitude less dose.

Overall, the RMSPE found for all three modalities is below the safety margins, which most particle therapy centers use to take uncertainties of the RSP prediction into account.^11^, ^31^, ^46^, ^47^

The gamma passing criteria (0.7%, 1mm, cf. Figure 3b) were based on the uncertainties Wohlfahrt et al.^27^ stated for the reference scan. Consequently, all passing pixels can be assumed to be in agreement with the reference. All three modalities were found to agree with more than 70% of their pixels with the reference scan, which is remarkable for the high‐density variations present in the imaged region. If the criterion is loosened to 2%, 2 mm or 3%, 3 mm, all modalities pass with approximately 95% and 99%, respectively, of the pixels.

The pixels with high relative deviations and high gamma values (cf. Figure 3) cluster in certain regions. The high (anti‐)correlation with some materials (cf. Figure 5) indicate that these cause high deviations in WET. The high correlation with air and sinus cavity explains the cluster of large deviations in the top of the WET difference map in Figure 3 for DECT‐DirectSPR and SECT‐HLUT. Here, a lot of voxels filled with air or sinus cavity tissue are present in the pixel channels and the overestimation of the RSP of these sums up to increased differences in WET. In contrast to that, voxels with bone, in particular cortical bone, seem to be underestimated by all modalities, which is demonstrated by a strong anti‐correlation between the percentage of voxels with cortical bone and the difference map. These observations are in line with the findings of Wohlfahrt et al.^27^ Here, SECT and DECT also show an overestimation of the RSP of air and sinus cavity tissue as well as an underestimation for cortical bone.

Naturally, the WET‐map of the reference scan is also connected to uncertainties. Wohlfahrt et al.^27^ validated the reference data set with multi‐layer ionization chambers and in that way obtained a range difference distribution, which is mostly comparable to our WET difference distribution. The RMSPE of this distribution^f^ can be estimated to be around 0.7 mm, which would correspond to 1.0% for their range. The MAPE determined analogously would be 0.8%. Thus, the MAPE and RMSPE of the reference amounts to approximately 60%– 80% of the ones determined for the three modalities presented in this work, indicating that these methods are capable of operating close to the limit of what is resolvable in a heterogeneous phantom.

Since in Figure 5b all imaging modalities show the same trend of over‐ and underestimation for the different materials, the WET measurement of the homogeneous slabs for the reference scan might as well contribute to the systematic deviations. Furthermore, the lower spatial resolution of the imaging modalities compared to the reference scan causes a smearing of structures. Therefore, the RSP is likely to be overestimated at the edge of features with low RSP values and vice versa.

To put the found WET deviations into perspective with previously published values, we first compare the WET accuracy of the αRad in this publication to the previous publication with the same imager. In a calibration phantom consisting of homogeneous plastic slabs, we reached a WET accuracy of below 0.3%.^23^ Taking into account that the WET accuracy is expected to be worse in more heterogeneous phantoms, the MAPE of (1.16 ± 0.05)% determined in this work is conceivable.

Because uncertainties in the RSP can add up or cancel out over the integration interval of the WET, these do not directly translate to WET uncertainty. Therefore, studies addressing RSP uncertainties are not suited for comparison to our method. Instead, we compare our values to range uncertainties in treatments planned on DECT and SECT images, which were published by several groups for anthropomorphic phantoms. These also contain the integration effects since the range equals the WET up to the stopping point of the ions. For a particle range of around 80—90 mm, Volz et al.^15^ reported a corresponding uncertainty of ‐1.40% (SECT) and ‐0.45% (DECT). Wohlfahrt et al.^27^ reached ‐0.3% to 0.1% (SECT) and 1.1% to 1.7% (DECT) for a similar particle range. These values were determined for different treatment plans in the head region with different fields and anatomies compared to our setup, but can still serve as a benchmark for the order of magnitude. Comparing our values to these, we can state that our reported deviations in WET are in line with these publications even though we partially dealt with greater depths (WETs of 93–173 mm) and still achieved similar accuracy.

In general, the found accuracy of both SECT‐HLUT and DECT‐DirectSPR are specific for the used HLUT and scanner at HIT since the inter‐center variability of RSP prediction was found to be above 2%.^48^

Comparing our result to the often cited range uncertainties in SECT images of 2.3%–2.6% (1.5 σ),^11^, ^12^ corresponding to 1.5%–1.7% if 1 σ is taken into account like it is done throughout this publication, the RMSPE found in this paper for SECT is rather small (1.30%) and surprisingly similar to the one of DECT.^49^, ^50^, ^51^ This could be caused by the fact, that a clinical HLUT with seven interpolated regions^52^ was used (following the consensus guide of Peters et al.^53^). Wohlfahrt et al.^27^ used three interpolated regions in their HLUT for imaging of the same anthropomorphic phantom. If our WET map based on SECT is reconstructed with a HLUT consisting of only four interpolated regions, the RMSPE worsens to 1.5%. The impact of the number of interpolated regions on the WET accuracy is observed here since the used anthropomorphic phantom consists of materials very similar to the ones used for the CT calibration procedure. Therefore, the RSP accuracy of these materials, which in contrast to patient tissue is close to the interpolated lines, benefits from a higher number of interpolated regions.

Before the presented ion‐beam radiography system can be used clinically, several aspects need to be addressed. The long imaging duration of around two hours for the presented radiograph is mostly caused by the dead time of the first generation Timepix detector: every measured frame (1 ms) is accompanied with 30 ms of dead time. Newer detector generations operate quasi dead time free due to a data driven readout instead of a frame‐based one. The imaging time could be drastically shortened by a factor of 30 due to the use of Timepix3^54^ or Timepix4^55^ detectors. Another aspect currently slowing down the imaging process is the limited field of view of the detector and the resulting subsequent acquisition of the ROIs. A synchronous movement of the detection system and a scanning beam could be a solution to increase the sensitive area above the current 2 ${\mathrm{cm}}^{2}$ at low cost. Alternatively, the Timepix 4 detector has a larger detector area (24.7 mm × 30.0 mm^55^) and can be tiled together at all four edges to obtain larger arrays, and therefore speed up the imaging process further. Together with the multi‐energy operation at HIT,^56^, ^57^ which could help to mitigate energy‐switching times, the stated factors could cause imaging times around a minute to become conceivable.

Additionally, the data post‐processing and image reconstruction has not yet been optimized for temporal performance. To render an online WET verification feasible, the current post‐processing time in the order of hours has to be also shortened towards less than a minute.

To be able to use our single‐ion tracking system for low‐dose imaging, a low intensity beam must be available. Currently, the particle flux has to be reduced to around 10–100 kHz. With Timepix3 or Timepix4, a data rate of around 1 MHz can be processed, which could fulfill the requirements for a clinically used system.^58^

We want to emphasize that we do not aim at replacing x‐ray CT imaging in the clinical workflow for ion‐beam radiotherapy. Our system is exclusively designed for radiographic imaging for regular WET verifications and not tomographic imaging, which is why treatment planning cannot be conducted based on our WET images. Furthermore, for our energy painting approach we need a prior WET estimate to choose the correct energy for the imaged region. Additionally, standard CT imaging techniques are significantly cheaper and faster.

However, the dose of x‐ray CTs usually is too high for daily imaging. Therefore, the authors think that the use of x‐ray CT imaging for treatment planning and αRad for regular verification of the patient anatomy is a very strong and promising combination for clinics.

On the day of each treatment fraction, several αRads, preferably along the field directions, could be acquired on couch directly before treatment starts. For opposing fields even imaging in one of the field directions is sufficient. In this way, movement uncertainties of the couch would also be ruled out and the patient anatomy can be verified in beams‐eye view. Given fast data processing, WET changes could directly be quantified, localized laterally, and thus their potential impact on the range of the ions could be estimated. Comparing the changes with the used safety margins would make decisions whether or not to trigger a control CT more sound. Moreover, coarse deviations of tissue RSP from the HLUT of the SECT could be detected, for example, in regions with several cavities. In case they exceed the combined uncertainty of αRad and SECT, a patient specific HLUT optimized by means of αRads with different field directions^59^, ^60^ could be a promising approach.

## 5 CONCLUSION

In this work, we presented the WET accuracy of the first helium‐beam radiograph of an anthropomorphic phantom acquired with thin silicon pixel detectors. It was shown to be capable of reaching a competitive WET accuracy compared to clinically established methods, namely, a single‐energy CT (SECT‐HLUT) and dual‐energy CT (DECT‐DirectSPR) scan, while depositing a dose on the order of a diagnostic x‐ray projection. Therefore, helium‐beam radiography could be a promising technique to not only detect, but also quantify anatomical changes in terms of WET on a daily basis. This can help to classify the impact of the change on the treatment plan of the patient. Thus, helium‐beam radiography emerges as a technique for regular ion‐beam range verification of ion‐beam radiotherapy treatments.

## CONFLICT OF INTEREST STATEMENT

The institution of Patrick Wohlfahrt and Christian Richter has a research agreement with Siemens Healthineers in the field of CT imaging for particle therapy. Furthermore, OncoRay has an institutional agreement as reference center for dual‐energy CT in radiotherapy as well as a software evaluation contract with Siemens Healthineers.

Jürgen Debus received grants or contracts with RaySearch Laboratories AB, Vision RT Limited, Merck Serono GmbH, Siemens Healthcare GmbH, PTW‐Freiburg, Dr. Pychlau GmbH, Accuray Incorporated. For the present study, the authors received no financial support from the sources listed in this section neither for the study design and materials, nor in the collection, analysis and interpretation of data nor in the writing of the publication.

All other authors do not have any conflicts of interest to disclose.

## Footnotes

a The stopping power of a certain material divided by the one of water is referred to as relative stopping power.

b purchased from ADVACAM s.r.o.

c Medical Information Toolkit (MITK), v2023.12 (ITK 5.3.0, VTK 9.2.6, Qt 5.12.12)

d Since the contrast of RSP data sets is worse compared to the original data sets showing CT numbers, the original data sets (pseudo mono‐energetic for DECT) were used for the image registration.

e based on an estimate using equation (5) of Amato et al.^35^ for the same setup. We used $D_{\text{ion}}=1.7\left(4\right)\;\mathrm{nGy}$ (see table II, 5.85mm).

f Figure 3, Half head Field A^27^ is the scenario which is mostly comparable to our setting.
